# Solar Energy Materials-Evolution and Niche Applications: A Literature Review

**DOI:** 10.3390/ma15155338

**Published:** 2022-08-03

**Authors:** Ntalane S. Seroka, Raymond Taziwa, Lindiwe Khotseng

**Affiliations:** 1 Department of Chemistry, University of the Western Cape, Robert Sobukwe Rd, Private Bag X17, Bellville 7535, South Africa; lkhotseng@uwc.ac.za; 2 Department of Applied Science, Faculty of Science Engineering and Technology, Walter Sisulu University, Old King William Town Road, Potsdam Site, East London 5200, South Africa; rtaziwa@wsu.ac.za

**Keywords:** solar cells, semiconductor, thin films, photovoltaic

## Abstract

The demand for energy has been a global concern over the years due to the ever increasing population which still generate electricity from non-renewable energy sources. Presently, energy produced worldwide is mostly from fossil fuels, which are non-renewable sources and release harmful by-products that are greenhouses gases. The sun is considered a source of clean, renewable energy, and the most abundant. With silicon being the element most used for the direct conversion of solar energy into electrical energy, solar cells are the technology corresponding to the solution of the problem of energy on our planet. Solar cell fabrication has undergone extensive study over the past several decades and improvement from one generation to another. The first solar cells were studied and grown on silicon wafers, in particular single crystals that formed silicon-based solar cells. With the further development in thin films, dye-sensitized solar cells and organic solar cells have significantly enhanced the efficiency of the cell. The manufacturing cost and efficiency hindered further development of the cell, although consumers still have confidence in the crystalline silicon material, which enjoys a fair share in the market for photovoltaics. This present review work provides niche and prominent features including the benefits and prospects of the first (mono-poly-crystalline silicon), second (amorphous silicon and thin films), and third generation (quantum dots, dye synthesized, polymer, and perovskite) of materials evolution in photovoltaics.

## 1. Introduction

Their contributions to sustainability and quality of life have led to the desire to develop, manufacture, and find new technologies based on renewable energy resources. Solar energy has proven to be sustainable and has attracted great attention, with the sun considered the most abundant source of clean, renewable energy. This makes solar cell technology economically viable and sustainable and allows for potential reductions in greenhouse gases, thus making it an ideal source of energy while avoiding shortcomings associated with energy and the environment [1,2,3,4].

**Figure 1:**
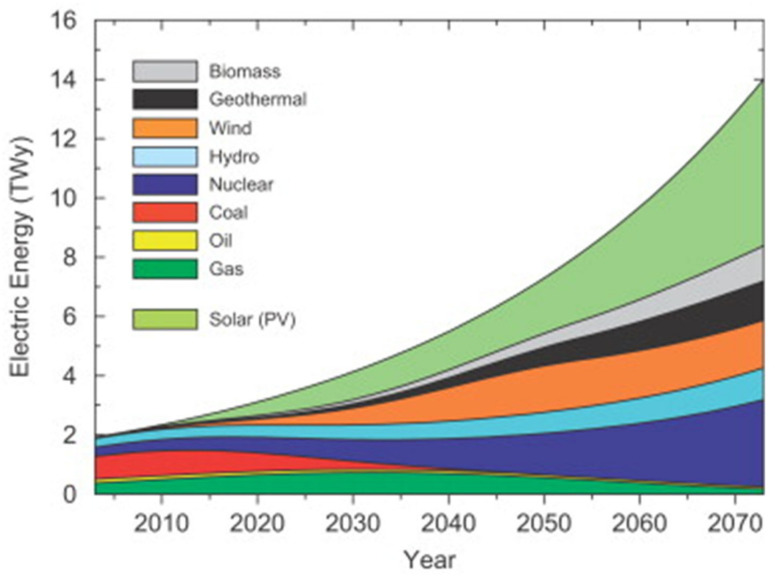
Presents electrical energy production for the next 6 decades (with consideration that the emission of CO_2_ will be held under 450 ppmv). Re-used with permission [5], Copyright © 2008 Elsevier.

Figure 1 presents the predicted and potential role of various sources in the future, with the stages of eco-friendly and renewable energy sources. The energy sources presented in Figure 1 show water, biomass, wind, solar, and geothermal energy. All these energy sources are environmentally friendly and sustainable. Of the aforementioned clean energy sources, solar technology promises to be the most prolific and fast-growing sustainable renewable energy source. [5].

The anticipation is solely based on the fact that the amount of CO_2_ will consistently and relatively remain below 450 ppm/year. Figure 1 significantly indicates that by 2050, solar energy is predicted to play a big role among the renewable sources in contributing to this [5,6,7].

Photovoltaics have the ability to generate electrical energy at a lower cost and they are eco-friendly. Thin film solar cells are favorable candidates in the field of photovoltaics because of their minimum material usage and rising efficiencies. The three most commonly known thin film technologies that are extensively studied and still under intensive investigation include α-silicon (α-Si), copper indium gallium selenide (CIGS), and star absorber materials, such as cadmium telluride (CdTe). The conventional inorganic silicon modules, interchangeably known as first generation solar cells, are the leading solar technology for the majority of residential and industrial markets. Presently, crystalline silicon wafers based on a high quality float zone have realized a competitive advantage over traditional solar cells via the utilization of the carrier-selective layer approach [8].

The amorphous silicon incorporated into an intrinsic hydrogen layer placed on one or two sides of the float zone silicon wafer serves as a passivation layer for pre-induced carrier-selective contact. Moreover, the aforementioned architecture recorded a power conversion efficiency of 26.6%, derived from the matrix of interdigitated back contact and heterojunction technologies, approaching the theoretical limit power conversion efficiency (PCE) of 29.1% for a silicon solar cell. In spite of its interesting characteristics and strong scalability for industrial and commercial use, the fabrication costs of this technology have halted, thereby making solar panels costly [8].

Thus, these drawbacks and limitations have led to the desire to research alternative materials such as (CIGS) and (CdTe), identified as second generation thin film photovoltaics, to adequately compensate for the inability of silicon photovoltaics to provide feasible manufacturing and production and generate inexpensive energy. Although crystalline silicon solar cells currently possess greater than 55% of the market share, the module efficiencies of CIGS and CdTe technologies almost rival that of crystalline solar cells, with recorded efficiencies of 21.4% and 21.6%, respectively [8,9,10,11].

Parallel with the emerging materials utilized in thin film technology, most focus is on perovskite solar technology and organic solar cells, as well as dye-sensitized solar cells (DSSCs). These plastic single-junction-based devices exhibit efficiencies of 25.5% [12], 16–18.1% [12,13,14,15,16] and 13% [12,17], respectively. These are third generation solar cells also known as plastic solar cells. Similarly, exfoliated tungsten telluride (WTe) flake (CiGSe) based multilayer thin films have been reported with a PCE of 10.87%. Furthermore, the structural manipulations of the active materials for these cells have garnered immense momentum due to their ease of production, flexibility, and simple fabrication methodologies [17,18].

Several studies have reported on these low-cost, complexity-free, and easily manufactured organic solar cells, more especially the π–conjugated polymers. The comprehensive scientific contribution towards the area of application-specific properties of π–conjugated polymers (viz., design, modelling, and fabrication) in the quest to match the electrical conducting properties exhibited by chemically doped polyacetylene is noteworthy. The aforementioned optoelectronic properties have opened technological interest in various applications, such as nano-electronics, sensors, internet of things (IOT), energy storage photodetectors, memory devices, field effect transistors (FETs), nonlinear optical devices, electrochromic, light emitting diodes (LEDs), and photovoltaics [18,19,20,21,22,23].

The conjugated polymers have interesting features owing to the side-chains present in the polymeric materials, which result in significant thermal stability and solubility, and ultimately promote the fabrication of these nanostructure-based materials by deposition with simple techniques, such as spin coating—hence, their inexpensive and simple solution processability. Their single- and double-bond characteristics and their interesting and adequate π-excessive nature have opened a window of opportunity for newly classified advanced materials in the arena of photonics and electronics [23,24].

For this study, we propose to utilize novel polycrystalline silicon semiconductor thin film raw material extracted from sugarcane bagasse ash (SCBA) for solar applications. Currently SCBA is being used as the source of fuel in power-generation boilers to produce electricity. Recent studies have shown that SCBA is rich with mineral content including silicon, iron, and other minerals. The silicon is found mainly in the form of silicic acid and constitutes about 380 kg/ha of silicon composition. It is worth noting that pure silicon is further obtained from silica (SiO_2_) and it has been reported that the silica is biocompatible and eco-friendly [25,26].

The disposal of bagasse ash waste, therefore, poses a dumping challenge to the environment. Currently, there is very little published information available on the production of silicon nanoparticles from sugarcane bagasse ash for solar applications. Furthermore, quartz is at present the main source of nano silicon. It is obtained by sand mining, which has detrimental effects on the environment such as land degradation, erosion, fissures, and adverse effects on water supply and quality. Metallurgical-grade silicon (MG-Si) (600,000 tons/year) is produced through high-temperature carbothermal reduction of quartz, which requires an energy input of 50 kWh/kg [26,27].

Herein, the three generations of solar materials are presented, including important parameters affecting the overall power output of the solar devices. The future prospects and challenges faced with current solar technologies are also discussed in detail.

## 2. Overview of Solar Cell

### 2.1. Solar Cell Principle of Operation

**Figure 2:**
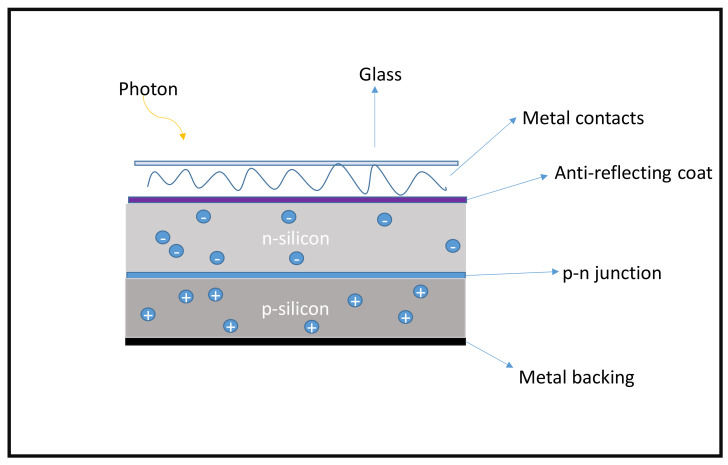
Schematic structure of typical a silicon solar cell [28].

Solar cells are mainly described based on their architecture; some consist mostly of metals (inorganic thin films), some nanomaterials (QD), some polymers (referred to as organic), etc. Traditionally, solar cells are electronic devices focused on converting sunlight into direct electrical energy as a consequence of the photoelectric effect from metals and inorganic semiconductors [28]. Figure 2 below shows a typical p-n junction silicon solar cell. The generation of electricity comes from the photons (light particles) when sunlight energy shines on the cell. Electrons (−) are ejected from doped Si (n-type) and move across to the positive (+) doped Si (p-type) material as illustrated in Figure 2 below.

#### 2.1.1. Electronic Structure and Doping Mechanisms in Crystalline Silicon

##### Electronic Structure

For the originality of electrical conductivity and advanced optical properties, namely low band-gaps in inorganics, it is critical to assimilate the reactivity of these materials, whose electronic configuration is exhibited by doping agents in comparison with undoped inorganic materials. It is well known that the electronic structure of silicon is [Ne] 3s^2^ 3p^2^, whereby valence electrons avail themselves to form four-bonds with neighboring (adjacent) atoms [29].

##### Doping Mechanisms of Silicon Materials

The tailoring of materials is performed to attain electrical properties and semiconductor characteristics via doping. This process involves the introduction of foreign materials and/impurities into the bare material, known as the intrinsic semiconductor, to successfully modulate its optical, electrical, and structural properties, ultimately labelled as the extrinsic semiconductor material. As a result of doping, chemical changes are induced within the silicon material, resulting in the generation of charges which migrate within the silicon matrix.

There are various ways and routes to carry out doping including redox, in situ, chemical (gaseous and solution), induced-radiation doping, and charge injection. Additionally, these can be further distinguished by the type of electron transfer, such as doping via reducing agent (n-type) or oxidizing agent (p-type) [29,30,31].

1. P-type Doping

Traditionally, silicon semiconductor doping involves the introduction of foreign materials such as boron and phosphorus to the silicon matrix. The foreign molecules and atoms are referred to as p- and n-type dopants, respectively, due to their electronic nature. Parallel to the concept of an inorganic semiconductor, charge creation emerges from the valence shell during the p-type doping process, as silicon is 1 e^-^ more and thus creates positive charges interchangeably referred to as holes, leaving the whole matrix with a positive charge. This process utilizes oxidizing agents such as boron [8,31].

2. N-type Doping

This process involves the generation of electrons via reducing agents such as phosphorus. In this case, referred to as n-type doping, the silicon matrix is altered as the reducing agent donates electrons, as illustrated in Figure 2, and the matrix generates a negative charge on the surface. N-type doping requires materials with an extra electron compared with the parent structure, silicon, as opposed to their counterpart p-type doping [31,32].

### 2.2. Important Parameters in a Solar Device

#### Principle of Charge Separation within a Solar Device

The concept of heterojunction cells relies on the efficient dissociation of an exciton, and the donor and acceptor material’s proximity factor plays a crucial role. The material thickness facilitates the optimum exciton diffusion length, usually a few tenths of a nanometer. Typically, the thickness of the active layer for organic semiconductors is in the range 80–200 nm. Recently, bulk heterojunctions have been deposited via co-sublimation of small molecules and/or the spin coating technique, using mixtures of polymers [33].

**Figure 3:**
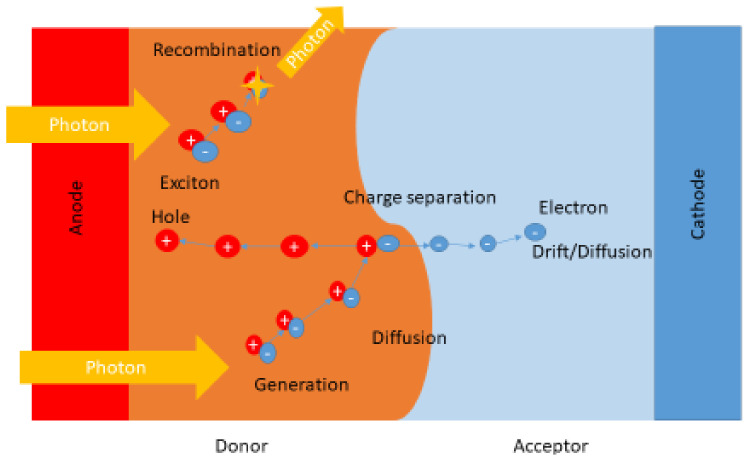
Illustrate the principle of charge separation within a solar cell [33].

The shortcomings faced in heterojunction structures derive from the fact that hole and electron transportation to the electrodes is required in order to promote the separation of charge carriers in order to reach their corresponding electrodes (cathode and anode). For instance, if the individual layer and a given bilayer structure are larger than that of the exciton diffusion length, it is most likely the excitons will recombine as shown in Figure 3, and result in the loss photon indicated by the exciton with the star. Although if the excitons generated are in close proximity to the interface, there is likelihood that they can be separated into free charge carriers and thus diffuse or drift towards their corresponding electrodes, as shown in Figure 3 [33,34,35]. 

**Figure 4:**
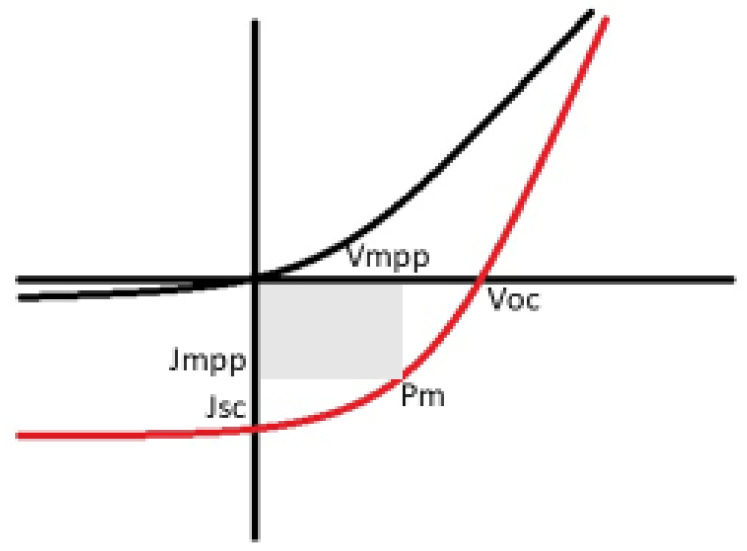
Represents a schematic current–voltage curve from a solar cell device black (dark) and red (under illumination) [36,37,38].

It is imperative to know the solar cell performance, as shown in Figure 4, which can be carried out by determining various factors including the fill factor, efficiency, short-circuit current density, and open-circuit voltage [36]. As a result of irradiation, the open-circuit voltage (*V_OC_*_)_ is cross-examined as the difference in the cell’s potential at the terminals when there is zero current flow through the terminals. Interestingly, the short-circuit current density *(J_sc_*) is produced from the cell upon irradiation at zero potential.

In addition, the fill factor *(FF*) is known to be the ratio between the cell’s maximum power, shown in Equation (1) where *V_mpp_* and *J_mpp_* denote voltage at the maximum power point and current at the maximum power point, respectively.

(1) $$FF=\frac{V_{mpp}J_{mpp}}{V_{oc}J_{sc}}$$

Ultimately the power conversion efficiency (PCE) is determined to be the ratio of the cell’s overall output power to incident radiant power.

(2) $$PCE=\frac{V_{oc}J_{sc}FF}{P_{in}}\times 100\%$$

**Figure 5:**
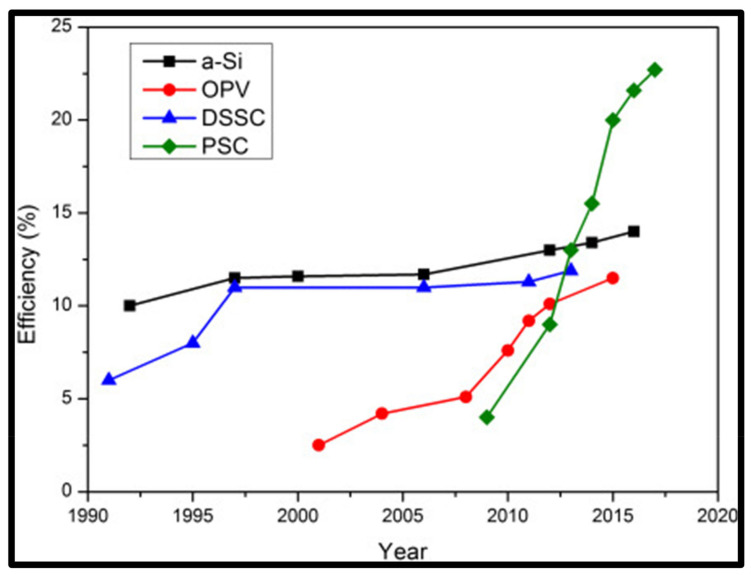
Progress of perovskite solar cell efficiencies compared with other thin film PV technology, reused with permission from [39], Copyright © 2018 Elsevier.

Innovative designs have made radical developments over the years, whereby thin film solar cell technology results in alternate material device structures. The rising efficiencies of thin film solar cells, in particular perovskite with 23% of the market share, have received significant attention in the photovoltaic market, mostly in the integrated photovoltaic (BIV) field. As shown in Figure 5, plastic solar cells (perovskite) have been rising in efficiency for over two decades, giving them potential as thin film PVs [39].

Thin film solar cells are favorable candidates in the field of photovoltaics because of their minimum material usage and rising efficiencies. The three most commonly known thin film technologies extensively studied and still under intensive investigations include α-silicon (α-Si), copper indium gallium selenide (CIGS), and star absorber materials such as cadmium telluride (CdTe). Photovoltaics have the ability to generate electrical energy at a lower cost and they are environmentally friendly. In spite of the aforementioned property (the power conversion from these devices with an enormous amount of the sun’s energy), these photovoltaic devices still produce about 30% or less of electricity from approximately 80–90% of the sun’s energy absorbed [39,40].

## 3. The First Generation Solar Cells

### 3.1. Crystalline Semiconductors

Crystalline materials are a solid material in which the structure’s dimensional patterns are consistently continuous throughout to the edges of the whole sample and remain unbroken. They are divided into different forms, i.e., monocrystalline, polycrystalline, etc. Monocrystalline simply means a single crystal with a grain-boundary-free uniform crystal lattice, while polycrystalline materials are essential in photovoltaics with majority-carrier bulk diffusion length.

Polycrystalline semiconductors are low cost materials relative to single-crystalline semiconductors. The first generation solar cells were built on silicon wafers. These are the most successful technology to date with high power efficacy. However, limitations result from their inhomogeneous nature and the reduced carrier bulk diffusion length of the material. This is a result of grain boundaries and dislocations as well as several other physical and chemical shortcomings with which we are faced with these materials. A grain boundary denotes a region in the material where adjacent atoms are disordered, consequently resulting in incomplete atomic bonding. These structural defects induce the trapping of electrons [41].

However, many attempts have been made to reduce the degree of grain boundaries formed in polycrystalline materials. Although polycrystalline semiconductors still lag behind in efficiency compared with monocrystalline solar cells, their tenable band gaps make them ideal materials, and this property can prove to be significant to the power conversion efficiency as well as to mitigate costs (modules) by utilization of low material costs [42].

#### 3.1.1. Nanomaterials

Nanoparticles are defined as the simplest form of structures with sizes in the nanometer range. In principle any collection of atoms bonded together with a structural radius less than 100 nm is essentially considered a nanoparticle. They differ from their bulk counterparts and isolated molecules owing to their chemical, electronic, and optical properties. The electronic properties of the materials are largely dependent on their dimensions and change dramatically as the density of states and the spatial length scale of the electronic motion are reduced with decreasing size. Therefore, size-induced changes in the electronic structure affect the optical properties of nanoparticles [43].

Inorganic semiconductors have revolutionized electronic devices owing to their excellent high-field-effect mobilities and long term stability far exceeding that of known organic materials. Thus they are conventional and the most commercialized types of electronic devices. Their main limitations are the material choice and fabrication strategies for the use of inorganic semiconductors, given that most polymer substrates are vulnerable to high operating temperatures required for traditional deposition methodologies, i.e., crystallization and doping. Researchers have reported on bottom-up approaches for inorganic semiconductors to produce zinc oxide (ZnO) nanowires. In addition, the dimensionality of these nanomaterials range from 1D and 2D with flexibility in portable electronics, as they possess outstanding optical, electrical, and mechanical properties [43].

#### 3.1.2. Conducting Polymers

The conducting polymers possess excellent ionic and electronic conductivity, optical transparency coupled with mechanical flexibility, rendering them the best lightweight flexible substrates. The transparent conductive oxide components normally used are indium tin oxide (ITO) and fluorine-doped tin oxide (FTO). As a result of material shortages, brittleness, and costs, conducting polymers are a potential alternative for TCO due to their excellent, simple nano/microscale self-assembly, abundancy, and mechanical flexibility [44,45].

The commonly studied conducting polymers include polypyrrole (Ppy), polythiophene (PT), polyaniline (PANI), poly(3,4-ethylenedioxythiophene) (PEDOT), and their derivatives. The materials mentioned above have promising applications in photovoltaics, thin-film transistors, supercapacitors, gas sensors, LEDs, and wearable electronics. Furthermore, their charge transport mechanisms are not well understood yet; it has been reported that intra chain charge transport enhancement exists as a result of new electronegative groups in conjugated backbones and acceptor dimerization, promoting the carrier mobility and stability of semiconducting polymers [44,45].

### 3.2. Single and Poly-Crystalline

Mono-crystalline silicon solar devices are composed of single crystals of silicon. Henceforth, the Si material is made with macro ingots, which results in Si crystallites (mono-crystalline Si) from the manufacturing process known as Czochralski [46,47,48].

The power efficiency is in the range 17–18% for the mono-crystalline silicon-based devices reported in [48]. However, due to the expensive and complicated process of forming single crystals, researchers have opted to modify the manufacturing processes. Molten silicon in a mold matrix of graphite produced polycrystalline silicon material. The as-prepared polycrystalline Si became the most popular solar cell worldwide since 2008, due to its economical properties, and constituted about 48% of solar cell production [49].

Although the solidification process of molten silicon produces a variety of crystal structures, and has proved to be cheaper to fabricate than monocrystalline silicon, it suffers very low efficiencies which lie between 12–14% [50].

## 4. Second Generation Devices (Thin Film Solar Cells)

In the quest to come up with alternative materials with superior electrical and optical properties, nanomaterials have been at center-stage for several applications in electronics, sensors, energy, etc. The ability to fine-tune their properties on the atomic and molecular level has caught the attention of many researchers worldwide. However, thin films have proved to be more viable and cost-effective.

### 4.1. Amorphous Silicon (α-Si)

The second generation α-Si, CIGS, and CdTe thin films, have been at center-stage as far as thin film solar cells evolution is concerned with Si still the star material in solar technology. The desired features of α-Si, such as its direct band gap, promote a fair fraction of sunlight to be absorbed into a thin layer of a few micrometers [51].

The amorphous Si is a material with low quality crystallinity, suffering from short order loose bonds temporarily resulting in minor carrier diffusion lengths with unfaceted electrical behavior. Further studies were conducted which attempted to improve on the aforementioned; hydrogen passivation (α-Si: H) proved to be a procedure for reducing loose bond densities by several orders of magnitude, thus improving the minority carrier length property [51].

The functionalization with H_2_ led to the light degradation known as Staebler–Wronski, whereby the efficiency of producing maximum electricity decreases. The optical band gap of α-Si: H of ≈ 1.7 eV can be fine-tuned to be more than 2 eV where maximum absorption starts. Moreover, it offers advantages such as shorter payback time (energy) and cost effective fabrication [8,51].

### 4.2. CdTe Thin Film

Since the 1950s, a great deal of attention has been placed on the development and increasing the efficiency of CdTe-based solar cells. Single-junction devices with an optimal band gap of 1.49 eV and a possible efficiency greater than 20% were desired for the CdTe solar cells to realize commercialization [52,53].

Furthermore, researchers reported on the first solar device with a significant conversion efficiency of 21.0% in 2014 [54]. Since then, CdTe thin film solar devices have realized a significant increase in efficiency to 22% [55]. However, this efficiency was not stable due to structural defects arising from grain boundaries and intra-grain dislocations. Another shortcoming was associated with a reduced lifespan for the minority carriers identified as one of the recombination factors, where it was presumably evident that the carriers recombine [56].

### 4.3. CIGS and CZTS Thin Films

Several decades ago, researchers extensively studied copper indium gallium selenide (GIGS) as one of the candidates for thin film absorber material. The scarcity of raw materials and the toxicity of these materials led to a breakthrough in alternative materials, namely copper zinc tin sulfur (CZTS). This material is an analogue of GIGS, preferably when Sn (IV), S (VI), and Zn (II) replace indium (III), Ga (III), and Se (Vi), respectively [57].

The opto-electronic as well as the structural properties of both the aforementioned thin films can be further improved by the displacement of their constituent element with readily available and earth-abundant compositions and toxin-free elements such as Sn, Sb, S_4_, and CuS [58]. For chemical treatment with hydrazine via non-vacuum particle solution, researchers have reported the highest efficiency of 12.7%, whereby the theoretical efficiency value was 32.4%. [59,60].

However, CZTS cells are prone to undesirably low voltages induced in the bulk material at the charge extraction interfaces as result of recombination defects. In spite of this, crucial breakthroughs in the development of CZTS-based solar cells rely on finding an alternative back contact with lower optical loss and maintaining low series resistance in rendering a high performance for the full device [61].

#### Tin Antimony Sulfide

In the quest to produce an environmentally friendly and earth-abundant binary compound, antimony sulfide (Sb2S_3_) has emerged as a potential candidate thin film material compared to toxic Cd or Pb. The material possesses a desired band gap of approximately 1.7 eV with a strong light extinction coefficient of 1.8 × 10^5^ cm^−1^ at 450 nm [62,63].

The abovementioned properties make antimony sulfide a suitable and ideal absorber material for thin film solar devices. Furthermore, the super efficiency reported for antimony-sulfide-sensitized solar cells has reached about 7.5% using a highly mesoporous device structure [64]. Several methods were utilized to fabricate the Sb_2_S_3_ layer, including spin coating [65], chemical bath deposition (CBD) [66,67] and successive layer adsorption [68]. The hole transport materials (HTMs) enhanced the photo carrier extraction for the as-fabricated solar cells [69].

Hence, the crystal quality of Sb_2_S_3_ determined the choice of HTMs, while some can poison the device stability in the long run, such as CuSCN, spiro-OMeTAD, or poly (3-hexylthiophene) (P3HT). The crystallinity of the absorber layer is crucial in this instance to avoid the utilization of HTMs. Further studies have revealed that the modification of planar solar cells based on TiO_2_/Sb_2_S_3_/P3HT planar solar cells has recorded 4.06% efficiency with the CBD method [70].

Therefore, the search to find the desired crystal quality of Sb_2_S_3_ and minimize decomposition continued; and the rapid thermal evaporation (RTE) method was adopted. Henceforth, an efficiency of 5.6% was achieved with the aforementioned method. Other methods were employed too, wherein, for traditional chalcogenide solar cells, annealing in selenium atmosphere was the preferred method to combat defect passivation and realize the improved crystallinity of Sb_2_S_3_ film. Consequently, sulfurization and selenization approaches were utilized to quench the oxides and passivate material defects [71].

## 5. Third Generation

These materials show far more promising breakthroughs in solar technology. Their application is yet to gain momentum as research is still ongoing for third generation materials in photovoltaic devices.

### 5.1. Quantum Dots (Nanocrystal Based)

Nanotechnology has been the a topic of interest in the science community for about a decade; various nanomaterials have been developed for several applications in electronics, sensors, the biomedical field, etc. Quantum dots (QDs) are nanoparticles of inorganic semiconductor materials. They usually have dimensions ranging from 1 nm to 10 nm which corresponds to 10–100 atoms. Their energy levels are quantized due to the confinement of electrons. The motion of electrons in the conduction band, valence band holes, and/exciton(s) is confined in all three spatial directions. Moreover, a variety of QDs have been traditionally produced from atoms in groups II-VI, III-V, and/IV-VI [72].

Quantum dot (QD) based photovoltaics absorb light from solution-processed nanocrystals and have versatile size-tunable band-gaps for fabrication in a wide range of substrates. These are a group of nanomaterials with good opto-electronic properties which are size-dependent. Therefore, the band gap of colloidal metal chalcogenide nanocrystals is invariably dependent on the size of the quantum dots. Consequently, this promotes efficient collection of near-infrared photons and the generation of multi-junction solar cells [73].

The quantum dot photovoltaics significantly benefit for their simple room temperature processing, fabrication, and air-durability operation. However, the present challenges faced within this field are the fundamental understanding of QD surface chemistry as well as their inherent disorder in quantum dot films due to mid-gap states which limit open-circuit voltages [74].

Although they have not yet realized large-scale industrial application, these technologies offer desired device properties, earth-abundance composition reliance, and simple processing techniques. Ideal QD solar cells have been fabricated using PbS and/or PbSe. Notably, they have the potential for novel applications in solar PV [75,76].

Another study reported on ligand free, methyl ammonium lead iodide (MAPbI_3_) QD solar cells with PCE above 9%. These materials were synthesized in situ within a porous silica (SiO_2_) matrix with a narrow nanopore size distribution. The synthesis parameter control translated to fine control over QD size with a spectral position on their electronic bandgap, preventing disorder that could affect the performance of the as-built devices [77].

In the classical sense of the word, a nanometer-sized semiconducting crystal confined in all three spatial dimensions is referred to as a quantum dot. The prevalent features of QDs include the generation of multi-excitons known as MEG and their tunability in optical and electronic states through reductions in size and quantum confinement effects. The MEG character in QDs allows the devices to rival that of a single junction Shockley–Queisser (S-Q) limit with 33% PCE. The three commonly used QDs extensively studied for the past 3 decades include CdTe, CdS/Se, and PbS/Se, and these are well represented in the light display market. With the resurgence of halide perovskite QDs, there is a potential new methodology for solar harvesting technology [78].

The practical conditions for perovskite QDs (PQD) are similar to that of traditional QDs, whereby organic capping ligands are utilized for growth control (steric hindrance) and new surface states. However, the nature of the surface leads to structural defects, and affects morphology and material stability. Colloidal QDs are ideal candidates for large-area device fabrication via printing techniques better suited than their counterpart organic and perovskite bulk counterparts. The highest PCE recorded for CsPbI_3_ QD-based solar cells was 16% better than that of PbS QDs, indicating a greater window of opportunity for next-generation QD photovoltaics [79,80].

### 5.2. Polymer Based Devices

Recently organic solar cells have attracted researchers as potential candidates for low cost energy conversion devices. For a single-junction configuration, these cells recorded a power conversion efficiency of 9.2% [81], which increased to over 12% [82] for a tandem structure. Another study focused on fullerene-derivative bulk hetero-junction (architecture) solar cells. The optimal thickness of 100 nm for this technology was noted from the most prominent features such as optical interference and recombination losses [81,82].

Additionally, organic materials generally have high absorption coefficient, although a considerable amount of light is lost due to transmission in semi-transparent and reflective electrodes on organic solar cells. Nevertheless, the efficacy of organic-based solar cells can be significantly improved to over 10% by understanding and mitigating such losses [83].

#### p-Conjugated Polymers as Hole-Transporting Layers (HTLS)

Since a breakthrough in 1958, solar cells have rapidly received a great deal of attention due to their material abundancy, inexpensiveness, flexibility, and ease of production. The processability of these materials has led to novel designs and device optimization as well as innovative nano-engineered architectures and single-junctions which relatively enhance the power conversion efficiencies to just over 17%, and approaching 18% [84,85].

M. H. Gharahcheshmeh et al., reported on the use of texture and nanostructure to enhance the electrical conductivity of semicrystalline conjugated polymers via water assisted (W-A) oxidative chemical vapor deposition (oCVD). The results revealed the charge transport between chains within the crystallite π–π stacking distance and interchain charge transfer integral. Interestingly, the use of W-A combined with a volatile oxidant, antimony pentachloride, recorded an electrical conductivity between 7520 ± 240 S cm^−1^, for PEDOT thin films [44,85].

The control on synthesis parameters for π–π stacking distance reduced from 3.50 Å down to 3.43 Å and recorded an electrical conductivity improvement of ≈1140 %. In addition, the highest electrical conductivity is also associated with a minimum Urbach energy of 205 meV, indicative of high morphological order. Another feature was the figure of merit (FoM) for transparent conductors, which reached an optimal maximum value of 94, 1.9× and 6.7× higher than oCVD PEDOT grown without W-A and usage of vanadium oxytrichloride and iron chloride agents, respectively. This procedure is cost-effective due to W-A oCVD being a single step all dry processes, involving direct growth of mechanical flexibility, conformal coverage, and rough structured surfaces with minimal to no complexity or costly transfer steps [44,85].

### 5.3. Dye Sensitized Based Solar Cells

Further studies were done especially on dye-sensitized solar cells (DSSCs) as emerging photovoltaic technologies. This technology has main components which work in synergy for the optimum performance of the cell, namely the redox electrolyte and photo-anode counter electrode. Several of the single and polycrystalline semiconductor materials used in the photo-electrodes include Si, InP, and GaAs [86].

DSSCs have good tunable optical properties (i.e., color and transparency), and are easy to fabricate as well as having low-cost manufacturing processes, and are lastly earth-abundant compositions with application-specific properties for photovoltaics. Their best recorded efficiency was only about 10% under irradiated sunlight. The main shortcoming of this technology is the choice of electrolyte which results in photo-degradation and destabilization of the cell, and thus a reduced lifespan [86].

Recent studies have reported on their improved power conversion efficiency of 14.3%. The stability of DSSCs has been relatively improved with time. Furthermore, with regard to light absorption and electron transport in particular it is imperative to find and develop photo-electrode materials for the betterment of DSSCs. Material suitability and applicability in solar applications have a huge effect on the power conversion efficiency. In addition, TiO_2_ is an ideal semiconductor photo-electrode material popular for DSSCs [87,88].

TiO_2_ nanomaterial exhibits superior textural properties such as high surface area (a large number of contact sides to promote the adsorption of dye molecules), and optimum electron transfer. It has been reported elsewhere that commercial TiO_2_ exhibits a band gap of 3.0–3.2 eV^9^ [89]

### 5.4. Perovskite Materials

Extensive research has continued over the years and a breakthrough has been made with recent polycrystalline films consisting of an organic cation (A), inorganic cation (B), and a halide (X) with the formula ABX_3_. The hybrid organic-inorganic material is commonly known as a perovskite material—henceforth, perovskite-based solar cells [90].

The desirable properties of perovskite materials include their long carrier diffusion length, band gap tenability, low recombination losses, and abundant material availability (low cost). These exceptional properties see PCEs reaching 25.5% efficiency, while enjoying low fabrication costs and facile synthesis as certified by the National Renewable Energy Laboratory (NREL). It is worth noting that the aforementioned are not available for traditional silicon-based solar cells [91]. However, the parasitic absorption in the back reflector and hole-conducting layer results in photocurrent loss for the perovskite absorption spectrum. In the quest to discover and develop perovskite technology, researchers reported on quasi-2D perovskite thin films, ${\left(C_{6}H_{5}{\mathrm{CH}}_{2}{\mathrm{NH}}_{3}\right)}_{2}{\left(\mathrm{FA}\right)}_{8}{\mathrm{Pb}}_{29}I_{28}$, where FA is formamidinium (FA), lead (Pb), and iodine (I), with excellent moisture resistance and a relative humidity of 80%, reaching a PCE of 17.40%. The moisture resistance is attributed to the ammonium salts. The recent discovery showed that 2D perovskite is versatile and can attain long-term stability as well as high efficiencies for PSCs [92,93].

The development of third generation perovskite solar cells has attained efficiencies of just over 20%. The continuous ability to fine-tune the band gap using Cl instead of I exhibited 3.2 eV from 1.6 eV, and incorporating low-band-gap materials improves the efficiency. Polycrystalline films are formed from the perovskite salts by precipitation methods with varying polar solvents. In 2019, Noh et al., tested a perovskite solar cell and attained 23.3% efficiency via an interface passivating method to minimize recombination at the interface [94].

Additionally, perovskite solar cells possess a significantly low fill factor of 0.73, which results from the non-uniform absorber (i.e., pinholes), and carrier shunt mechanisms caused by carrier-selective contacts (resistive losses associated with non-ideal carrier-selective contacts). Most importantly, severe environmental stability issues limit the industrial application of these materials. It is worthwhile to appreciate the possible market entry-point in which perovskite serves as a top cell in Si/perovskite tandem cells with a fundamentally large band gap [95].

## 6. Outlook

Greener technology involves a moderate methodology with non-hazardous chemicals and the utilization of organic acids and bases, resulting in significant time and energy savings while also allowing the synthesis of high-value compounds for specified applications. Green procedures set out to create no harmful by-products and allow tailoring for more precise control of particle shape, size, and appearance in the nano-realm. Bio-nanoparticle production is an environmentally friendly, low-cost, long-term approach for extracting high-value nano-silicon directly from naturally occurring agricultural wastes such as sugarcane bagasse, where Si is found in the form of ${\mathrm{SiO}}_{2}$ [96,97].

### Future Prospects and Challenges

**Figure 6:**
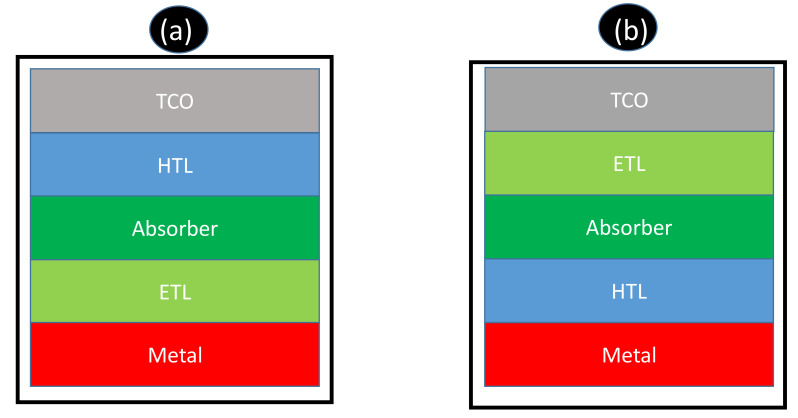
Schematic representation a (**a**) typical solar cell and (**b**) inverted solar cell architecture.

Figure 6 below presents scheme(s) for a conventional (a) and inverted (b) solar cell device structure. It is possible to observe that the two proposed structures consist of several layers including transparent conductive oxide (TCO), a hole-transporting layer (HTL), an absorber layer, an electron transporting layer (ETL), and a metal. It is imperative to emphasize that these layers serve different functions synergistically to produce current from charge transportation induced by incident light [98].

Transparent conducting oxide(s) are conventionally chosen as an anti-reflective coater (ARC), as well as the conducting anodic electrode on the front side of the cell. Normally, TCO layers with a thickness in the range 70 to 150 nm in silicon-based solar cells are desired to promote adequate transmission of light to the absorbing material and minimize optical losses. Researchers reported more than 90% minimal sheet resistance and smooth surface morphologies when the TCO layer, mostly indium tin oxide (ITO) is between 80 to 180 nm [96,97,98].

Metallization, has found to be effective at the rear/back side of the cell, namely gold (Au), aluminum (Al), and silver (Ag). Interestingly, Gwamuri et al. [98] successfully reported that an ITO thickness of about 50 nm produces transmittance of approximately 80% with higher sheet resistance data, which is not desired for solar technology. Thus, the threshold minimum of 70 nm must be corroborated in order to find synergism between smooth morphologies, sheet resistivity, and transmittance. Recommendations:

➢ Use alternative sources of solar materials, such biomass, to minimize costs.
➢ Use green technology, such as biomass (eco-friendly), a renewable source for solar materials.
➢ Use hybrid organic-silicon heterojunction solar cells.
➢ Employ solar-grade silicon from silica (metallothermic reduction as compared to carbothermal reduction process).
➢ Enormous scientific research efforts in the past were devoted to the development and optimization of the following;
➢ ETL, HTL, perovskite composition, thickness, process, and device structures.
➢ The state-of-the-art perovskite and non-toxic solar cells will lead to the development and discovery of new Pb-free perovskite light absorber materials which are environmentally friendly and critical in the field of PVs. The new research area will be imperative in realizing stable and eco-friendly perovskite PVs for real-world applications.

## 7. Summary

Crystalline silicon solar cells have been used for many decades. Although this technology is at its maturity stage, it is still not financially feasible for developing countries due to the high cost of raw materials and the large surface area required. Solar cell research based on heterojunctions (donor–acceptor) with staggered electronic band alignment, named the type-II configuration, has the potential to address efficiency limitations in already existing technologies. In spite of this, it is in still in its mature research stage and its application might be attained in the near future. Thin film technology is presently producing suitable candidates for application-specific properties where there are non-permanent structures, such as there being no flat roof in buildings, and this is a niche market for these solar cells. Interestingly, the efficiency of thin film solar cells has improved over the years when raw materials are utilized. Silicon remains the most commercialized, and hybrid architecture allows for the maximum potential of utilizing this star material as well as looking for alternative sources to eventually minimize the fabrication costs of these devices.
